# Dual anti-HER2/EGFR inhibition synergistically increases therapeutic effects and alters tumor oxygenation in HNSCC

**DOI:** 10.1038/s41598-024-52897-5

**Published:** 2024-02-14

**Authors:** Patrick N. Song, Shannon E. Lynch, Chloe T. DeMellier, Ameer Mansur, Carlos A. Gallegos, Brian D. Wright, Yolanda E. Hartman, Laura E. Minton, Suzanne E. Lapi, Jason M. Warram, Anna G. Sorace

**Affiliations:** 1 Department of Radiology, The University of Alabama at Birmingham, 1670 University Blvd, Birmingham, AL 35233 USA; 2 Graduate Biomedical Sciences, The University of Alabama at Birmingham, Birmingham, USA; 3 Department of Biomedical Engineering, The University of Alabama at Birmingham, Birmingham, USA; 4 Department of Otolaryngology, The University of Alabama at Birmingham, Birmingham, USA; 5 O’Neal Comprehensive Cancer Center, The University of Alabama at Birmingham, Birmingham, USA

**Keywords:** Head and neck cancer, Molecular medicine, Oncology

## Abstract

Epidermal growth factor receptor (EGFR), human epidermal growth factor receptor 2 (HER2), and hypoxia are associated with radioresistance. The goal of this study is to study the synergy of anti-HER2, trastuzumab, and anti-EGFR, cetuximab, and characterize the tumor microenvironment components that may lead to increased radiation sensitivity with dual anti-HER2/EGFR therapy in head and neck squamous cell carcinoma (HNSCC). Positron emission tomography (PET) imaging ([^89^Zr]-panitumumab and [^89^Zr]-pertuzumab) was used to characterize EGFR and HER2 in HNSCC cell line tumors. HNSCC cells were treated with trastuzumab, cetuximab, or combination followed by radiation to assess for viability and radiosensitivity (colony forming assay, immunofluorescence, and flow cytometry). In vivo, [^18^F]-FMISO-PET imaging was used to quantify changes in oxygenation during treatment. Bliss Test of Synergy was used to identify combination treatment synergy. Quantifying EGFR and HER2 receptor expression revealed a 50% increase in heterogeneity of HER2 relative to EGFR. In vitro, dual trastuzumab-cetuximab therapy shows significant decreases in DNA damage response and increased response to radiation therapy (p < 0.05). In vivo, tumors treated with dual anti-HER2/EGFR demonstrated decreased tumor hypoxia, when compared to single agent therapies. Dual trastuzumab-cetuximab demonstrates synergy and can affect tumor oxygenation in HNSCC. Combination trastuzumab-cetuximab modulates the tumor microenvironment through reductions in tumor hypoxia and induces sustained treatment synergy.

## Introduction

Head and neck squamous cell carcinoma (HNSCC) encompass cancers of the oral cavity, pharynx, salivary glands, and nasal cavity and is the sixth most common cancer with a 50–60% 5-year survival rate^1,2^. Head and neck squamous cell carinomas encompass a wide range of organs, therefore the 5-year survival rate varies depending on location. For example, early laryngeal cancers have a 60% 5-year survival rate and oral cavity cancers have a 70% 5-year survival rate, whereas late-stage laryngeal cancers have a much lower survival at 34%^3^. Due to a wide range of causal factors (alcohol, tobacco, and HPV status), HNSCC represents a highly heterogenous tumor subtype. Tumor heterogeneity has been shown in HNSCC in hypoxia, immune cell infiltration, DNA repair response, and cellular proliferation^4–6^. This increased intratumoral heterogeneity results in a complex tumor microenvironment^7^. These changes in tumoral phenotype drive differences in intratumoral response to first-line radiotherapy^8–10^. First-line treatment of HNSCC remains surgical, however patients with inoperable tumors or regional metastasis must undergo a combination of radiation therapy and chemotherapy^11^. Due to the innate radiosensitivity of the head and neck region and the high incidence of off-target toxicity, there is demand for treatment regimens that improve radiotherapeutic sensitivity, while decreasing overall radiation exposure^12^.

As 90% of HNSCC overexpress the gene for the epidermal growth factor receptor (EGFR), a targeted antibody therapy to EGFR, cetuximab, has been examined in combination with radiation therapy and has been observed to decrease locoregional tumor burden and reduce mortality^1,13–15^. While cetuximab has been showed to improve tumor control, it has also been shown to increase radiosensitivity of surrounding normal tissue to radiotherapy side effects^14,15^. Bonomo et al*.* observed that a frequent side effect of combination cetuximab and radiation is severe radiation dermatitis, highlighting the difficulties in implementing combination cetuximab and radiation for widespread use^14,15^. Up to 19% of HNSCC also overexpress the human epidermal growth factor receptor 2 (HER2) gene, thereby revealing an interesting opportunity to examine anti-EGFR and anti-HER2 dual treatment synergy to reduce HNSCC tumor burden. Further targeting of HER2 has shown to synergize with radiotherapy in cancers that overexpress HER2, therefore providing an opportunity to investigate these effects in HNSCC^16^. As HNSCC can potentially express both receptors, this provides an avenue for evaluating if dual EGFR/HER2 inhibition can sensitize HNSCC to low doses of radiation therapy.

Non-invasive advanced imaging techniques, such as positron emission tomography (PET) can identify changes in intratumoral molecular biology that drives phenotypic changes in therapeutic response^16–26^. Lu et al*.* used non-invasive [^89^Zr]-pertuzumab PET imaging to monitor baseline HER2 expression and noted a positive correlation between baseline HER2 expression and response to paclitaxel therapy, opening the possibility to predict eventual response to cytotoxic therapy^21^. [^18^F]-Fluoromisonidazole (FMISO) hypoxia PET imaging has previously been used to monitor anti-HER2 trastuzumab induced changes in intratumoral hypoxia to guide fractionated radiotherapy in mouse models of primary HER2+ breast cancer^16^. These results found that image-guided radiotherapy induced longitudinal sustained tumoral response independent of additional therapy. Due to the inherent heterogeneity of HNSCC, there is a demand for novel therapeutic strategies for improving prognosis of patients with HNSCC undergoing therapy.

The overall goal of this study is to characterize HNSCC for EGFR and HER2 expression and evaluate if trastuzumab and cetuximab can synergize and enhance radiosensitivity in HER2+/EGFR+ HNSCC. We hypothesize that longitudinal in vitro cell viability studies and in vivo PET imaging can identify states of enhanced susceptibility to radiation therapy through changes in tumor oxygenation. This study incorporates: (1) immunofluorescence and novel PET imaging to assess EGFR and HER2 expression in HNSCC, (2) in vitro live cell imaging of treatment synergy in three HNSCC cell lines, (3) PET imaging of hypoxia in response to combination HER2-targeted and EGFR-targeted therapies and (4) in vivo monitoring of longitudinal tumor changes in response to combination trastuzumab and cetuximab therapy. This provides an approach to better understand the relationship of EGFR and HER2 in HNSCC and its role in altering hypoxia and therapeutic response to radiation therapy.

## Materials and methods

### Cell culture

HNSCC cell lines were purchased from American Tissue Type Collection (Manassas, VA). SCC1 (Cellosaurus, accession number CVCL_7707), FADU (Cellosaurus, accession number CVCL_1218), and OSC19 (Cellosaurus, accession number CVCL_3086) were cultured at 37 °C, 5% CO_2_ and maintained in Dulbecco’s Minimal Essential Medium (Thermofisher Scientific, Waltham, MA, USA) supplemented with 10% FBS, 2 mM l-glutamine and 1 mM sodium. All cells were maintained to 70–90% confluency before passaging.

### Molecular probing of receptor expression with immunofluorescence

To study the spatial distribution of HER2 and EGFR, SCC1, FADU and OSC19 cells were fixed, probed with rabbit anti-HER2/ErbB2 (Cell Signaling Technology catalog #: 2242) and mouse anti-EGFR (Thermofisher catalog #: MA1-24678), and stained with TRITC donkey anti-rabbit (Jaxson labs catalog #: 711-025-152) and Alexa Fluor 647 donkey anti-mouse (Jaxson labs catalog #: 715-605-150) and counterstained with DAPI (Southern Biotech catalog #: 0100-20). Cells were subsequently imaged with the EVOS M7000 with DAPI (Excitation/emission: 357–444/447–460 nm), RFP (Excitation/emission: 531–540/593–640 nm) and Cy5 (Excitation/emission: 628–640/692–740 nm) fluorescent channels. Quantification of HER2 and EGFR expression was assessed through Celleste Image Analysis software (Thermofisher Scientific, USA). Co-localization analysis allowed for assessing of HER2+ signaling through co-localization of RFP and DAPI). Co-localization analysis allowed for assessing of EGFR+ signaling through co-localization of Cy5 and DAPI. Co-localization analysis allowed for assessing of HER2+ and EGFR+ signaling through co-localization of RFP, Cy5 and DAPI, respectively.

Quantification of HER2 and EGFR expression was assessed through Celleste Image Analysis software (Thermofisher Scientific, USA). Co-localization analysis allowed for assessing of HER2+ signaling through co-localization of RFP and DAPI). Co-localization analysis allowed for assessing of EGFR+ signaling through co-localization of Cy5 and DAPI. Co-localization analysis allowed for assessing of HER2+ and EGFR+ signaling through co-localization of RFP, Cy5, and DAPI, respectively.

### In vitro model

For in vitro live cell imaging experiments, SCC1, FADU and OSC19 cell lines were transfected to express green fluorescent protein (GFP) for longitudinal tracking of cell viability. The plasmid and protocol used to induce stable nuclear transfection were previously described^27^. Briefly, cell lines were co-transfected with pCMV (CAT) T7-SB100 (Addgene plasmid #34879) and a GFP clone Sleeping Beauty compatible vector (Addgene plasmid #60525). Cells were selectively cultured and separated with fluorescence activated cell sorting.

GFP-SCC1, GFP-FADU and GFP-OSC19 cells were plated on 96-well plates (Fisher Scientific catalog #: 3596) at a density of 10,000 cells/well and were imaged every 24 h with the EVOS M7000 imaging system (ThermoFisher, Waltham, MA, USA) at 4× magnification. Fluorescent images were quantified with automated custom MATLAB image analysis code to quantify viability as a function of fluorescent objects (available upon request).

#### Evaluation of treatment synergy in vitro between trastuzumab and cetuximab

To evaluate treatment synergy between trastuzumab and cetuximab in HNSCC cell line models, cells were treated with trastuzumab, cetuximab, or a combination of trastuzumab and cetuximab. On day 0, cells (10,000 cells/well) were plated and, 24 h later, cells were treated with control, trastuzumab (100 µg/mL), cetuximab (250 µg/mL) or a combination of trastuzumab and cetuximab and treatment was sustained until the endpoint of the experiment (day 5). Each group had five replicates.

#### Modulation of anti-HER2/EGFR synergy in HNSCC in vitro

To identify driving mechanisms of synergy between trastuzumab and cetuximab, doses of trastuzumab and cetuximab were modulated to induced varying states of HER2 or EGFR inhibition and subsequent response was evaluated. On day 0, HNSCC cell lines (10,000 cells/well) were seeded on a 96-well plate and, 24 h later, were treated with media-only control, trastuzumab (50 or 100 µg/mL), cetuximab (250 or 500 µg/mL), or a combination trastuzumab and cetuximab, and imaged for four days (Supplemental Table 1). Each group had four-six replicates.

#### Combination anti-HER2/EGFR inhibition and radiation in HNSCC in vitro

To evaluate the potential of HER2/EGFR inhibition for radiosensitization, HNSCC cell lines were treated with trastuzumab, cetuximab and a combination of trastuzumab and cetuximab prior to fractionated radiation therapy. On day 0, HNSCC cell lines (10,000 cells/well) were plated and, 24 h later, were treated with a combination of trastuzumab and cetuximab. Cells were then irradiated with 2 Gy on day 2, 3, and 4 with the X-RAD 320 irradiator (Precision X-ray, North Bradford, CT, USA) at 320 kV power and 12.5 mA current. Cells were monitored for an additional two days following final radiation fraction before experimental endpoint. Each group had five replicates.

#### Clonogenic survival assay to monitor combination synergy and radiosensitivity

Longitudinal cancer cell line viability in response to radiation therapy was assessed with a colony forming assay. SCC1 or FADU cells were seeded in a 12 well plate at a density of 1000 cells/well and treated with 2 Gy radiation, 100 µg/mL trastuzumab, 250 µg/mL cetuximab or a combination of trastuzumab and cetuximab and radiation. Following treatment, cells grew undisturbed for two weeks. After two weeks, cells were washed, fixed, stained with 0.01% crystal violet (Fisher Scientific Catalog # AC40583025, Waltham, MA, USA), and air dried. Well plates were imaged (EVOS M7000). Experiments were performed with four replicates per experimental condition. To quantify clonogenic assays, images were automatically quantified through QuPath Machine Learning Algorithm. Colonies with less than 50 cells were excluded from analysis. Overlapping colonies were separated with ImageJ watershed thresholding.

#### Molecular probing of DNA repair immediately following combination dual anti-HER2/EGFR and radiotherapy

Longitudinal DNA damage and DNA damage repair was assessed with molecular probing of phospho-γ H2AX and Rad51, respectively. On day 0, SCC1 cells (10,000 cells/well) were plated on a 96 well plate and, 24 h later, treated with 100 µg/mL trastuzumab, and 250 µg/mL cetuximab. On day 2, targeted therapy was removed, and cells were irradiated with 2 Gy radiotherapy. Following radiotherapy, cells were immediately trypsinized and fixed. Cells were stained with 1:200 mouse anti-phospho-γ H2AX (Cell Signaling catalog #: 80312S) or 1:200 mouse anti-Rad51 (Thermofisher Catalog #: MA1-23271) and 1:500 Alexa Fluor 647 donkey anti-mouse. Cells were then analyzed with an Attune NxT Flow Cytometer to probe for phospho-γ H2AX and Rad51 expression.

### Tumor model

All animal experiments were approved by The University of Alabama at Birmingham’s Institutional animal care and use committee and were performed in accordance with relevant animal guidelines and regulations. This study is in accordance with ARRIVE guidelines. Five to six-week-old female athymic nude mice were obtained from Charles River Laboratories (catalog number: 490) and subcutaneously engrafted with 10^7^ SCC1 or FADU cells in serum free DMEM + 30% Matrigel in the right shoulder of the mouse. Tumors were monitored on a weekly basis and enrolled into the study when tumors were approximately 100–200 mm^3^. Tumor outside of this range were not considered clinically relevant and were excluded from experiments. For treatment studies, tumors were randomly sorted into treatment groups. For all animal procedures, mice were anesthestized at 2% isofluorine. Tumor bearing mice were randomized prior to start of experiment.

#### [^89^Zr]-pertuzumab and [^89^Zr]-panitumumab PET imaging to assess intratumoral distribution of HER2 and EGFR in HNSCC tumors

To determine the spatial distribution and heterogeneity of HER2 and EGFR in HNSCC tumors, mice were engrafted with FADU tumors, injected with [^89^Zr]-pertuzumab and [^89^Zr]-panitumumab, and imaged with PET/CT imaging. Pertuzumab (Medchemexpress, Catalog #: HY-P9912) and Panitumumab (Amgen, Thousand Oaks, CA, USA) were conjugated with 0.4 mg deferoxamine (DFO) at a concentration of 10 mg/mL. ^89^Zr was purchased as a service by The University of Alabama at Birmingham’s cyclotron facility. DFO-Pertuzumab or DFO-panitumumab was labeled with [^89^Zr] at 6 µCi/µg and approximately 66.8 ± 15 µCi of [^89^Zr]-pertuzumab (n = 6) or 76 ± 22.5 µCi of [^89^Zr]-panitumumab (n = 3) was injected intravenously. Approximately 10–12 µg of radiolabeled antibody was administered prior to imaging. Five days post radiotracer injection, mice were imaged for 20 min with static ^89^Zr-PET imaging followed by a 5 min CT, at 80 kVp, with an integrated small animal PET/CT (Sofie Biosciences, Somerset, NJ, USA). Tumor regions of interest were manually delineated using CT anatomical imaging. Mean standardized uptake value (SUV) and intratumoral distribution of [^89^Zr]-pertuzumab and [^89^Zr]-panitumumab uptake was quantified (VivoQuant, InviCRO, Boston, MA). Quantification of the Full Width at Half Maximum (FWHM) was used to assess the distribution of SUV intratumoral heterogeneity of HER2 and EGFR expression.

#### FMISO-PET imaging to monitor dual HER2 and EGFR inhibition effect on hypoxia

To determine whether dual HER2-EGFR inhibition improves molecular tumor oxygenation and induces a state of increased radiation sensitivity in vivo, SCC1 tumors (N = 3–4 per experimental condition) were treated with either 10 mg/kg trastuzumab, 30 mg/kg cetuximab or a combination of trastuzumab and cetuximab and imaged with static [^18^F]-FMISO-PET imaging. FMISO was synthesized by The University of Alabama at Birmingham’s cyclotron facility as a service and was generated on a GE FASTlab2 or Synthra RNplus module. Mice with SCC1 tumors (N = 12, N = 3 per condition) were imaged with [^18^F]-FMISO-PET on days 0, 5, 7 and 14. At each timepoint, mice were injected with approximately 150 µCi (149.4 ± 10.3 µCi) of FMISO intravenously and imaged for 20 min with [^18^F]-FMISO-PET imaging at 80 min post-injection followed by CT. PET images were processed as previously described.

#### Longitudinal monitoring of tumor viability in tumors treated with combination trastuzumab and cetuximab therapy

To determine whether trastuzumab and cetuximab synergistically improves tumor inhibition and cytotoxicity in vivo, SCC1 (N = 3 per experimental condition) and FADU (N = 3–4 per experimental condition) tumors were treated with saline control, 5 mg/kg trastuzumab, 5 mg/kg cetuximab or a combination of trastuzumab and cetuximab via IP injection on day 0 and 3. Tumors were then monitored three times per week for an additional four weeks for changes in longitudinal tumor viability.

### Statistical analysis

Experimental conditions were summarized by average of replicates and error was represented by standard error of mean (SEM). A parametric T-test was used to assess significant difference between groups. A Grubbs outlier test was used to eliminate any replicates that were statistical outliers. All data and figures were analyzed using GraphPad Prism 7 (La Jolla, CA, USA). The Bliss test of synergy was used to quantify synergy between therapies^28^.

### Ethics approval and consent to participate

All procedures were approved by our institution’s animal care and use committee under APN 21611.

## Results

### Molecular probing of HNSCC cell lines reveals heterogeneous expression of HER2 and EGFR

**Figure 1:**
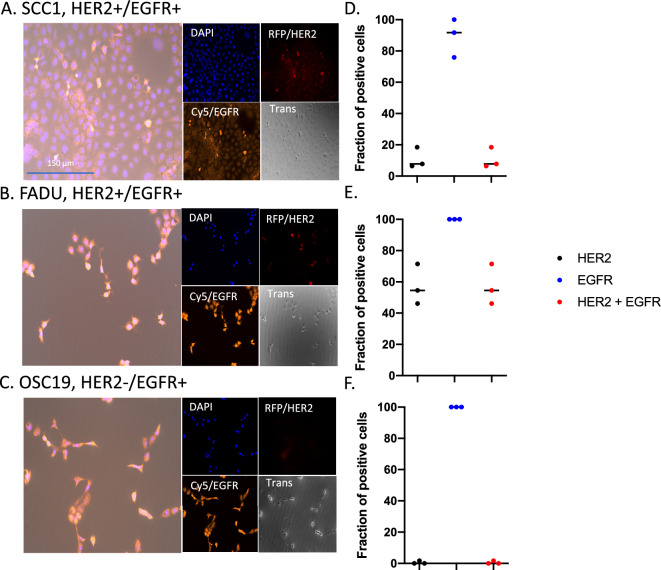
Molecular probing of HER2 and EGFR in HNSCC cell lines with immunofluorescence in vitro. SCC1 (**A**), FADU (**B**) and OSC19 (**C**) cell lines were probed with anti-HER2, anti-EGFR and DAPI. Quantification of HER2, EGFR and both expression of HER2 and EGFR in SCC1 (**D**), and FADU cells (**E**) and only EGFR expression in OSC19 cells (**F**).

**Figure 2:**
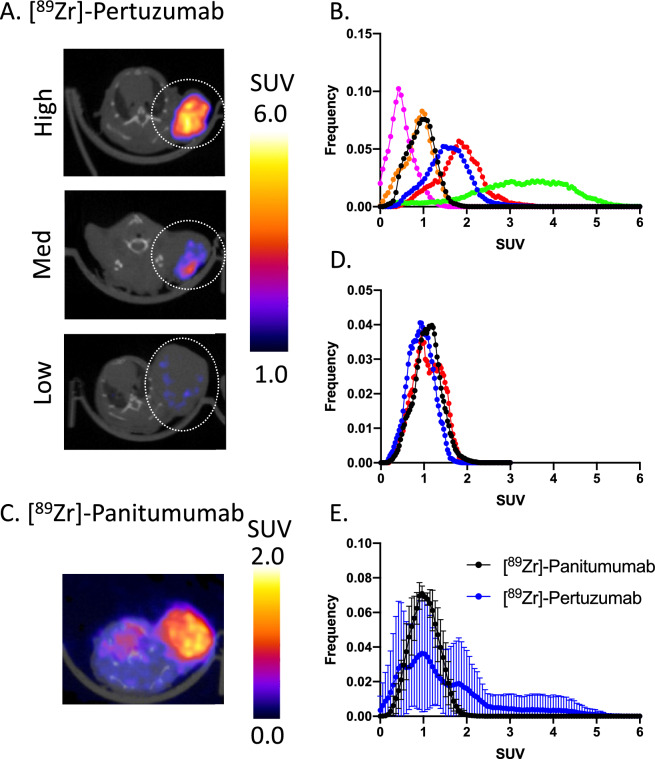
In vivo probing of HER2 and EGFR in FADU tumors with immune-PET imaging. FADU tumors were injected and imaged with [^89^Zr]-pertuzumab PET imaging (**A**) and quantified for HER2 expression. Individual tumor distribution of [^89^Zr]-pertuzumab was quantified (**B**) and averaged (**E**). FADU tumors were imaged with [^89^Zr]-panitumumab (**C**) and quantified for EGFR expression. Individual tumor distribution of [^89^Zr]-panitumumab was quantified (**D**) and averaged (**F**). A full width at half maximum test (FWHM) was used to study the heterogeneity of HER2 expression.

In vitro, SCC1 and FADU reveal heterogeneous distribution of HER2 and EGFR (Fig. 1A,B, respectively); whereas, OSC19 reveals heterogeneous distribution of EGFR but no expression of HER2 (Fig. 1C). All cells demonstrating HER2 have expression of EGFR as well. SCC1 cells show 10.9 ± 6.6% and 89.2 ± 12.2% of the cells are positive for HER2 or EGFR, respectively (Fig. 1D). FADU cells show 57.4 ± 12.9% and 100% of the cells are positive for HER2 or EGFR, respectively (Fig. 1E). OSC19 cells show 0.6 ± 1.0% and 100% of the cells are positive for HER2 or EGFR, respectively (Fig. 1F). In vivo, FADU tumors were imaged with [^89^Zr]-pertuzumab (HER2) and [^89^Zr]-panitumumab (EGFR) (Fig. 2A,C) and heterogeneity (as shown with histogram of the SUV of all tumor voxels) of uptake was quantified. FADU tumors had a [^89^Zr]-panitumumab SUVmean of 1.04 ± 0.09 and evaluation of the histogram of SUV frequency revealed a FWHM of 0.85 (Fig. 2B,D); whereas, FADU tumors had a [^89^Zr]-pertuzumab SUVmean of 1.48 ± 0.97 and a FWHM of 1.28 (Fig. 2E), suggesting a 50.5% greater distribution of HER2 than EGFR.

### Combination trastuzumab and cetuximab significantly decreases tumor proliferation relative to single agent targeted therapy

**Figure 3:**
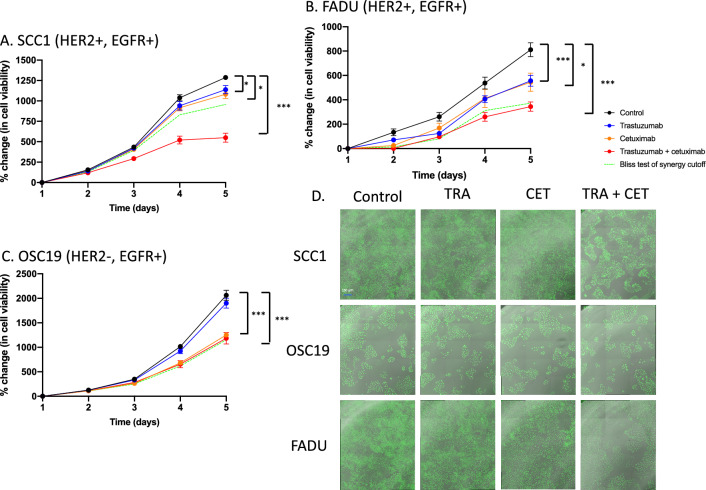
Quantitative live cell imaging of trastuzumab and cetuximab interaction in vitro. SCC1 (**A**), FADU (**B**) and OSC19 (**C**) cell lines were treated with combination trastuzumab (100 µg/mL) and cetuximab (250 µg/mL) and synergy was tested between anti-HER2 and EGFR therapy. A synergistic relationship was observed in HER2+ EGFR+ cell lines; however, an additive relationship was observed in HER2− EGFR+ cell lines. Representative images of SCC1 and OSC19 show the impact of combination trastuzumab and cetuximab dual inhibition (**D**).

Figure 3 shows in vitro live cell imaging of SCC1, FADU and OSC19 cells in response to dual trastuzumab-cetuximab therapy. On day 5, SCC1 cells treated with trastuzumab or cetuximab alone show 1138.4 ± 111.8% (p = 0.03, relative to control) and 1082.4 ± 119% (p < 0.01, relative to control) growth, respectively. However, SCC1 cells treated with trastuzumab and cetuximab dual therapy show a 549.8 ± 124% (p < 0.0001, relative to control. p < 0.001, relative to cetuximab treated) growth relative to control (Fig. 3A,D). This trend was also observed in the FADU (HER2+/EGFR+) cell line. FADU cells treated with trastuzumab or cetuximab alone show a 555.9 ± 98.9% (p < 0.01, relative to control) or 544.3 ± 166.8% growth relative to control, respectively. FADU treated with trastuzumab and cetuximab dual therapy exhibited 344.2 ± 94.9% growth, relative to control (p < 0.0001, relative to control. p = 0.03, relative to cetuximab treated) (Fig. 3B). Conversely, there was no synergistic therapuetic benefit from adding trastuzumab to the OSC19 (HER2−/EGFR+) treated with cetuximab (trastuzumab and cetuximab dual therapy exhibited an 1184.9 ± 234.6% growth compared to 1250.8 ± 72.5% growth when treated with cetuximab alone (p = 0.61 relative to cetuximab treated) (Fig. 3C,D). Bliss test of synergy confirms synergistic effect between trastuzumab and cetuximab in HER2+/EGFR+ SCC1 and FADU cell lines and additive effect of trastuzumab and cetuximab in the HER2−/EGFR+ OSC19 cell line.

### Modulation of combination trastuzumab and cetuximab reveals synergy is driven by increased HER2 inhibition

**Figure 4:**
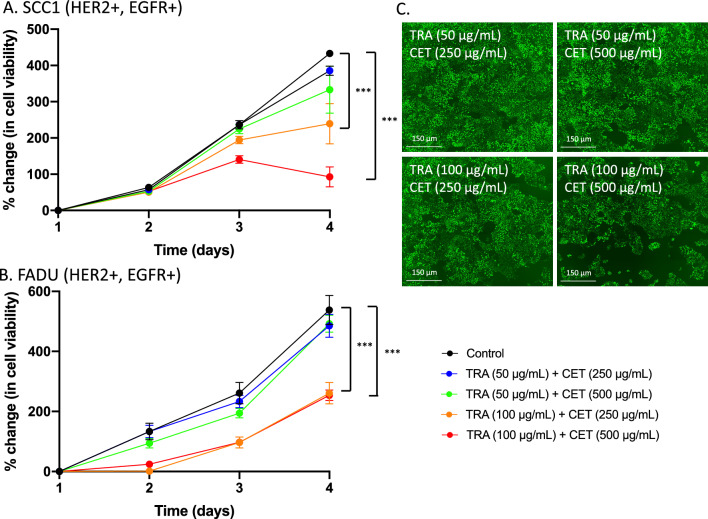
Modulation of treatment synergy in vitro reveals treatment synergy is driven by enhanced HER2 inhibition. SCC1 (**A**) and FADU (**B**) cell lines were treated with varying doses of trastuzumab (50 µg/mL or 100 µg/mL) or cetuximab (250 µg/mL or 500 µg/mL) and synergy was only identified in groups with increased trastuzumab dosing. Representative images of SCC1 and FADU show the impact of synergy modulation on cell viability (**C**).

Figure 4 shows HER2 inhibition drives synergy in trastuzumab-cetuximab treated cells. When treated with 50 µg/mL trastuzumab, SCC1 cells experienced a 385.6 ± 28.0% (p = 0.02, relative to control) and 333.1 ± 144.2% (p = 0.22, relative to control) growth, when simultaneously treated with 250 µg/mL or 500 µg/mL cetuximab, respectively. When treated with 100 µg/mL trastuzumab, SCC1 cells experienced a 239.5 ± 124.6% (p = 0.02, relative to control) and 92.5 ± 55.6% (p < 0.0001, relative to control) when simultaneously treated with 250 µg/mL or 500 µg/mL cetuximab (Fig. 4A). When treated with 50 µg/mL trastuzumab, FADU cells experienced a 485.4 ± 93.6% (p = 0.41, relative to control) and 492.4 ± 68.8% (p = 0.42, relative to control) increase in cell growth when simultaneously treated with 250 µg/mL or 500 µg/mL cetuximab, respectively. When treated with 100 µg/mL trastuzumab, FADU cells experienced a 260 ± 87.3% (p < 0.01, relative to control) and 253.5 ± 43.8% (p < 0.001, relative to control) growth when simultaneously treated with 250 µg/mL or 500 µg/mL cetuximab (Fig. 4B). Increased growth inhibition observed at high concentrations of trastuzumab, irrespective of cetuximab concentration, suggest combination synergy is primarily driven by HER2 inhibition (Fig. 4C).

### Dual trastuzumab-cetuximab improves radiosensitivity in HER2+/EGFR+ HNSCC

**Figure 5:**
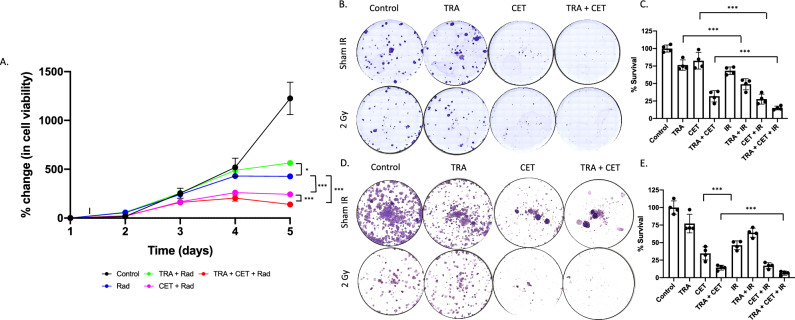
Combination trastuzumab-cetuximab treated groups improves cytotoxicity of radiotherapy irrespective of cetuximab. SCC1 or FADU cells were treated with a combination of trastuzumab (100 µg/mL), cetuximab (250 µg/mL) and radiotherapy (2 Gy) and viability was monitored (**A**). Increased cytotoxicity was observed irrespective of cetuximab dose. A colony forming assay was also used to confirm impaired DNA damage response in cells treated with dual trastuzumab-cetuximab prior to radiotherapy (**B**) and quantified (**C**) in SCC1 cells or FADU cells (**D** and **E**).

Figure 5 demonstrates dual anti-HER2/EGFR potentiates response to radiotherapy in HER2+/EGFR+ HNSCC (Fig. 5A). Control SCC1 cells experienced 1225.9 ± 371.3% growth on day 5. When treated with radiotherapy, SCC1 cells experienced 426.8 ± 58.3% (p < 0.01, relative to control) growth on day 5. SCC1 cells treated with trastuzumab prior to radiotherapy experienced 564.7 ± 59.8% growth (p < 0.01, relative to control) and cells treated with cetuximab prior to radiotherapy experienced 243.3 ± 25.3% growth on day 5 (p < 0.01, relative to control). When treated with trastuzumab + cetuximab + radiation, cells experienced 139.8 ± 69.9% growth on day 5 (p < 0.01, relative to control). Colony forming assays confirmed long term growth inhibition (Fig. 5B,D), showing that dual anti-HER2+ and anti-EGFR inhibition (trastuzumab + cetuximab) consistently improved radiation cytotoxicity and resulted in the greatest inhibition of growth kinetics through colony forming assay and longitudinal treatment response. In both SCC1 and FADU cell lines, the cells treated with trastuzumab + cetuximab + radiation therapy consistently exhibited the greatest inhibition in colony formation compared to combination treated groups (p < 0.01) (Fig. 5C,E).

### Molecular probing of DNA damage reveals anti-HER2/EGFR delays DNA damage repair through reduced NHEJ

**Figure 6:**
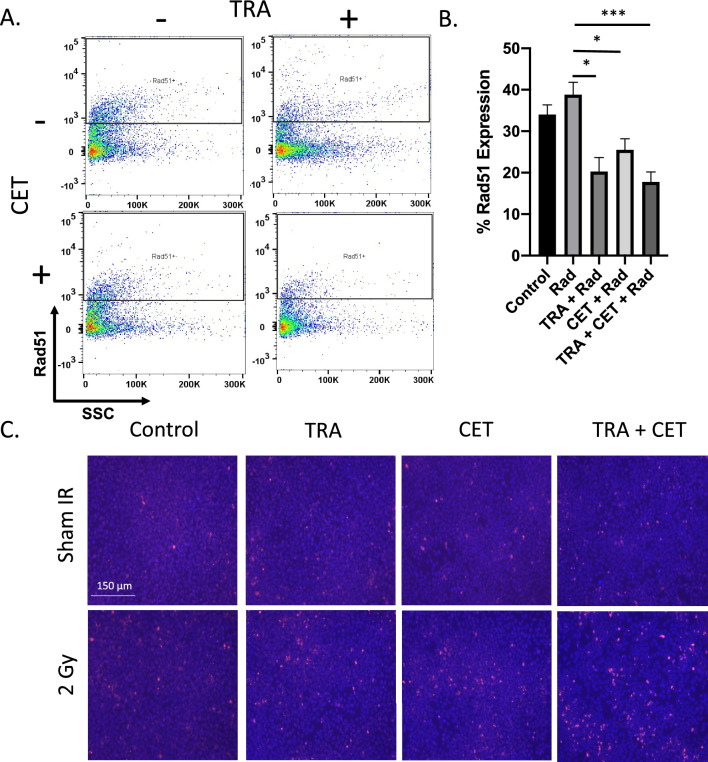
Mechanistic probing of synergy reveals significant modulation of the cellular and molecular microenvironment to sensitize to radiotherapy when treated with dual HER2− EGFR therapy. Flow cytometry probing Rad51 (**A**, **B**) was used to examine changes in DNA damage response in cells treated with trastuzumab (100 µg/mL), cetuximab (250 µg/mL) and radiation (2 Gy) and was quantified. Qualitative phospho-γ H2AX was conducted on HER2+/EGFR+ HNSCC cell line, which shows increased expression when pre-treated with anti-HER2 and anti-EGFR therapy prior to radiation therapy (**C**).

Figure 6 shows changes in the cellular microenvironment in response to dual anti-HER2/EGFR to sensitize cells to longitudinal treatment response. To examine molecular changes in DNA damage response, SCC1 cells were treated with 100 µg/mL trastuzumab and 250 µg/mL cetuximab prior to radiation therapy and probed against Rad51. SCC1 cells treated with 2 Gy radiation experienced a 39.5% expression of Rad51. When treated with trastuzumab prior to radiation therapy, SCC1 cells experienced a 20.8% expression of Rad51 (p = 0.01, relative to radiation treated). When treated with cetuximab prior to radiation therapy, SCC1 cells experienced a 25.5% expression of Rad51 (p = 0.02, relative to radiation treated). When treated with trastuzumab and cetuximab prior to radiation therapy, SCC1 cells experienced a 17.8% expression of Rad51 (p < 0.01, relative to radiation treated) (Fig. 6A,B). Results suggest combination anti-HER2 and anti-EGFR therapy may sensitize HNSCC to long term radiation sensitivity.

To identify mechanisms of enhanced response to combination trastuzumab, cetuximab, and radiation, cells were treated and given two days to recover nonspecific DNA damage prior to immunofluorescent probing. Qualitative IF staining revealed increases in phospho-γ H2AX after two days of DNA damage repair in replicates that were treated with trastuzumab and cetuximab prior to radiotherapy. Cells that were pre-treated with targeted anti-HER2 and anti-EGFR therapy show substantial increases in DNA damage several days after irradiation (Fig. 6C).

### FMISO-PET imaging reveals significant decreases in tumor hypoxia in trastuzumab and combination treated tumors

**Figure 7:**
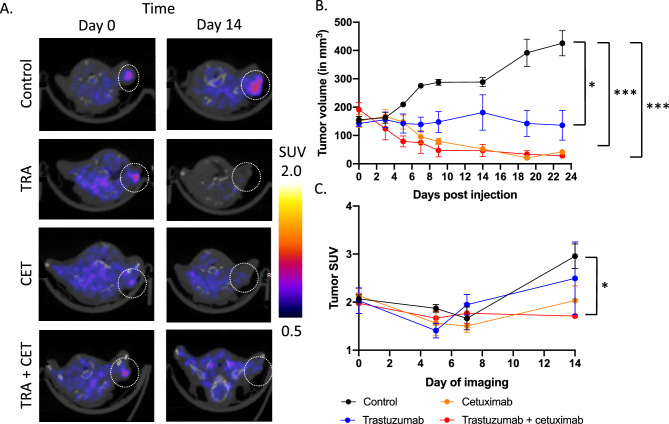
FMISO-PET imaging of combination trastuzumab-cetuximab treated tumors reveals significant modulation of the tumor microenvironment to sensitize to radiation therapy. SCC1 tumors were treated with trastuzumab (10 mg/kg) and cetuximab (30 mg/kg) and were imaged with FMISO-PET imaging on day 0, 5, 7 and 14 (**A**). Significant changes in tumor viability were observed in SCC1 tumors treated with trastuzumab, cetuximab or a combination of trastuzumab and cetuximab (**B**). Significant changes in tumor hypoxia were observed in trastuzumab-cetuximab combination treated groups (p = 0.04) (**C**).

Figure 7 demonstrates decreases in the hypoxic tumor microenvironment in response to trastuzumab and cetuximab combination therapy in HER2+/EGFR+ HNSCC tumors (Fig. 7A). SCC1 tumors treated with vehicle control had a tumor volume of 425.72 ± 62.6 mm^3^, while tumors treated with trastuzumab or cetuximab single agent therapy had a 68.1% (p < 0.05) or 90.2% (p < 0.01) decrease in growth, relative to control. SCC1 tumors treated with combination trastuzumab and cetuximab had a 93.4% (p < 0.01) decrease in growth, relative to control (Fig. 7B). SCC1 tumors treated with vehicle control had a FMISO SUV of 2.96 ± 0.36, while tumors treated with trastuzumab or cetuximab single agent therapy had a FMISO SUV of 2.50 ± 1.32 (p = 0.68, relative to control) or 2.03 ± 0.53 (p = 0.12, relative to control). SCC1 tumors treated with combination trastuzumab and cetuximab had a FMISO SUV of 1.71 ± 0.07 (p = 0.04, relative to control) (Fig. 7C).

### Longitudinal tumor monitoring reveals sustained decreases in tumor volume in response to nontherapeutic dual trastuzumab + cetuximab therapy

**Figure 8:**
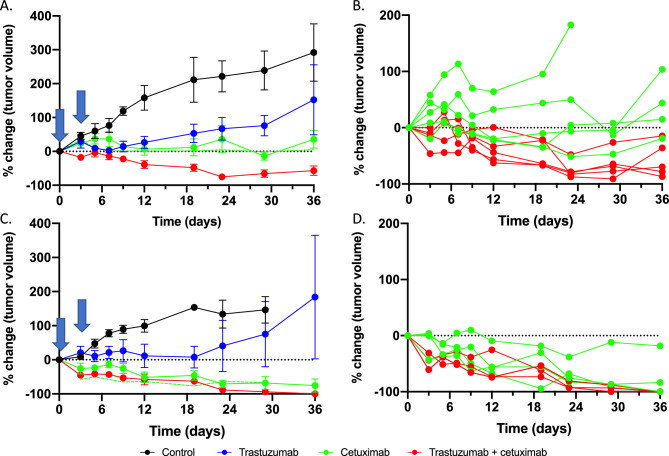
Low dose treatment synergy of trastuzumab-cetuximab treated HNSCC tumors reveals substantial reductions in tumor burden after two doses of therapy. FADU (**A**) and SCC1 (**B**) tumors were treated with two doses of trastuzumab (5 mg/kg), cetuximab (5 mg/kg) or a combination and longitudinal response was monitored. Significant and sustained treatment response was observed four weeks following therapy. Sustained decreases in tumor viability were observed in FADU tumors treated with trastuzumab + cetuximab (**C**) and 100% complete tumor response was observed in SCC1 tumors treated with trastuzumab + cetuximab, whereas 33% complete tumor was observed in SCC1 tumors treated with cetuximab (**D**).

Figure 8 reveals longitudinal sustained response in HER2+/EGFR+ HNSCC tumors treated with low dose combination trastuzumab and cetuximab therapy. FADU tumors treated with trastuzumab or cetuximab monotherapy experienced a 47.9% (p = 0.37) or 87.7% (p = 0.02) decrease in growth, relative to control. FADU tumors treated with combination trastuzumab and cetuximab therapy experienced a 123% (p < 0.01) decrease in tumor volume, relative to control (Fig. 8A). Among individual treated tumors, 25% of cetuximab treated tumors decreased in tumor volume by day 36; however, 100% of combination trastuzumab-cetuximab treated tumors decreased in tumor volume by day 36 (Fig. 8B). SCC1 tumors treated with trastuzumab or cetuximab monotherapy experienced a 25.7% increase in tumor volume, relative to control (p = 0.87) and 151% decrease in tumor volume, relative to control (p < 0.01). SCC1 tumors treated with combination trastuzumab and cetuximab therapy completely responded to therapy with 100% of tumors responding to therapy (p < 0.01, relative to control) (Fig. 8C). Furthermore, while cetuximab treated SCC1 tumors completed responded at a rate of 50%, combination trastuzumab-cetuximab therapy increases the rate of complete response to 100% of treated tumors (Fig. 8D).

## Discussion

Targeted EGFR therapies in combination with radiation therapy for HNSCC have been well characterized and implemented into clinical practice; however, therapeutic efficacy and off-target effects remain a challenge. As normal tissue of the head and neck region innately express EGFR, novel receptor targeting strategies are warranted to improve intratumoral radiosensitivity, while decreasing overall EGFR targeting. These studies demonstrate how the addition of anti-HER2 therapy can improve overall radiosensitivity while allowing for dose de-escalation of anti-EGFR therapy. In this study, we used in vitro longitudinal live cell imaging, clonogenic assays, flow cytometry, immunofluorescence, in vivo hypoxia imaging and longitudinal tumor monitoring to elucidate the impact of dual HER2 and EGFR inhibition on cellular and molecular mechanisms of radiosensitivity. In vitro, we observed that trastuzumab synergizes with cetuximab at the cellular level in HER2+ EGFR+ HNSCC and, by modulating the dose of trastuzumab, we identified that this synergy is primarily driven by HER2 inhibition, although further study of synergistic interactions between IC25 and IC50 should be investigated. A Bliss test of synergy was utilized to identify synergy between trastuzumab and cetuximab; however, as the Bliss test of synergy utilizes the average effect at each time point, further statistical modeling of synergy may be useful to understand the temporal interactions of anti-HER2 and anti-EGFR therapies. In vivo, we identified that combination trastuzumab-cetuximab decreases tumor hypoxia and can induce a state of longitudinal sustained response after two doses of trastuzumab (5 mg/kg) and cetuximab (5 mg/kg). To our knowledge, this study is the first to examine heterogeneity of HER2 expression in HNSCC and to study the dual targeting of HER2/EGFR in HNSCC in the context of radiosensitization; however, our studies are supported by research into dual HER2/EGFR inhibition in pancreatic and gastrointestinal cancer^29–36^. It does not appear as though heterogeneity of HER2 expression in these models is related to changes in tumor growth, as there was no correlation in tumor volume. Larbouret et al*.* observed that pancreatic tumor bearing mice demonstrated significant increases in survival when treated with anti-HER2/anti-EGFR dual regimens in comparison to standard first-line treatment, gemcitabine^32^. Similarly, Zheng et al. found combination trastuzumab and cetuximab re-sensitizes trastuzumab resistant gastric cancer to anti-HER2 therapy^33^. As a number of cancers overexpress both HER2 and EGFR (i.e. gastric, ovarian, breast), our results have potential to be translated into a wide range of solid tumors and PET imaging has potential to be used to identify which patients would be susceptible to dual HER2/EGFR combination therapies.

The radiosensitization of combination anti-HER2/anti-EGFR synergy studies build upon previous studies into hypoxia and DNA damage modulation in HER2+ cancer^16,19,26^. In other studies, trastuzumab doses up to 10 mg/kg were used to simulate clinical response and were observed to modulate the tumor microenvironment^19,22,24,37–39^. Song et al. observed that a subtherapeutic dose of trastuzumab (4 mg/kg) significantly reduced tumor hypoxia by 30% and increased innate immune cell activation when combined with radiotherapy in HER2+ primary breast cancer^16^. In this study, we observed the synergy of trastuzumab and cetuximab in vitro and in vivo. Dual inhibition of HER2 and EGFR provides an approach to reduce tumor cell growth signaling cascades. Using immunofluorescence and FMISO-PET imaging, we identify that combination trastuzumab and cetuximab radiosensitizes HNSCC through delayed DNA damage repair and decreased hypoxia. Future studies can examine how sequential treatment strategies can affect cell signaling compensation, cell signaling preferences and cell synchronization by probing for changes in signaling with western blot.

Differences in response to dual anti-EGFR and HER2 therapy in HNSCC in vitro and in vivo may be attributed to differences in human epidermal growth factor compensatory pathway signaling^40^. In SCC1 tumors, we observed 100% response to combination trastuzumab-cetuximab, whereas in FADU tumors, we observed 50% response. Zhang et al*.* notes that HER3 is upregulated in response to dual anti-HER2/EGFR tyrosine kinase inhibitor therapy^41^. SCC1 and FADU cell lines have starkly different expression of HER3, which may explain differences in response to combination trastuzumab-cetuximab therapy^42,43^. Analysis of growth rates and their effect on the temporal variations in cell receptor expression may play a role in these drug relationships and should be further investigated. Because synergy was observed with trastuzumab doses of 100 ug/mL irrespective of cetuximab dosage, it is suggested that combination trastuzumab-cetuximab synergy is driven primarily by anti-HER2 inhibition. Interestingly, FADU cells have higher levels of HER2 expression compared to SCC1 cells, yet clonogenic assays deonstrated significant increases in radiosensitization in trastuzumab + radiation treated SCC1 cells and no significant increase in radiosensitization in trastuzumab + radiation treated FADU cells (Supplementary Table 1).

To further evaluate the clinical applications of our study, it would be beneficial to study the immune interaction between dual anti-HER2/EGFR synergy. To study the impact of T-cells and B-cells on the synergy and radiosensitization of dual trastuzumab and cetuximab combination therapy, experiments conducted in a humanized model of HER2+/EGFR+ HNSCC could be pursued. Similarly, future directions could explore additional alterations when utilizing a HNSCC orthotopic tumor engraftment. Zeng et al*.* studied the impact of combination prexasertib and cisplatin on the radiosensitization of Luc + SCC1 cell injected into the tongue^44^. While this is important to explore, PET imaging approaches in orthotopic tumor preclinical models of this size would provide challenges (while still being feasible for clinical translation). Follow-up experiments into dual HER2/EGFR inhibition could examine the impact of HER2 and EGFR small molecular inhibitors (such as lapatinib, tucatinib and gefitinib) and whether synergy and radiosensitization are still observed through decreased DNA damage response and hypoxia in vitro and in vivo, respectively. Finally while cetuximab efficacy has been studied in the context of an IC50 concentration in HNSCC, trastuzumab’s IC50 concentration in HER2 + HNSCC should be evaluated^42,45^.

## Conclusions

This study reveals preliminary evidence that dual HER2 and EGFR inhibition synergizes in HNSCC to induce a state of longitudinal therapeutic synergy in vitro and in vivo. For in vitro and in vivo tumor models, combination trastuzumab and cetuximab pre-treatment increased DNA damage (increased phospho-γ H2AX and decreased Rad51) and decreased tumor hypoxia. These results highlight the potential for combination anti-HER2 and anti-EGFR inhibition in HNSCC and in cancers that overexpress both HER2 and EGFR (i.e. ovarian cancer and trastuzumab resistant HER2+ breast cancer) to decrease tumor proliferation, improve overall patient prognosis, and inform on overall clinical decision making through a combination of low, nontherapeutic doses of therapy.

### Supplementary Information

Supplementary Legends.

Supplementary Table 1.

## Data Availability

Datasets and materials are available upon reasonable request to the lead contact, Anna Sorace (asorace@uabmc.edu).

## References

[1] Zeng L (2017). Combining Chk1/2 inhibition with cetuximab and radiation enhances in vitro and in vivo cytotoxicity in head and neck squamous cell carcinoma. Mol. Cancer Ther..

[2] Vigneswaran N, Williams MD (2014). Epidemiologic trends in head and neck cancer and aids in diagnosis. Oral. Maxillofac. Surg. Clin. N. Am..

[3] Hoesseini A (2020). Physicians' clinical prediction of survival in head and neck cancer patients in the palliative phase. BMC Palliat. Care.

[4] Canning M (2019). Heterogeneity of the head and neck squamous cell carcinoma immune landscape and its impact on immunotherapy. Front. Cell Dev. Biol..

[5] Elmusrati A, Wang J, Wang CY (2021). Tumor microenvironment and immune evasion in head and neck squamous cell carcinoma. Int. J. Oral Sci..

[6] Economopoulou, P., I. Kotsantis, & A. Psyrri. Tumor microenvironment and immunotherapy response in head and neck cancer. *Cancers (Basel)*. **12**(11) (2020). 10.3390/cancers12113377 PMC7696050 33203092

[7] Picon H, Guddati AK (2020). Mechanisms of resistance in head and neck cancer. Am. J. Cancer Res..

[8] Alterio D (2019). Modern radiotherapy for head and neck cancer. Semin. Oncol..

[9] Hakansson K (2020). Radiation dose-painting with protons vs photons for head-and-neck cancer. Acta Oncol..

[10] Alfouzan AF (2021). Radiation therapy in head and neck cancer. Saudi Med. J..

[11] Brook I (2020). Late side effects of radiation treatment for head and neck cancer. Radiat. Oncol. J..

[12] Pryor DI (2009). Enhanced toxicity with concurrent cetuximab and radiotherapy in head and neck cancer. Radiother. Oncol..

[13] Bonner JA (2010). Radiotherapy plus cetuximab for locoregionally advanced head and neck cancer: 5-year survival data from a phase 3 randomised trial, and relation between cetuximab-induced rash and survival. Lancet Oncol..

[14] Kanakamedala MR, Packianathan S, Vijayakumar S (2010). Lack of Cetuximab induced skin toxicity in a previously irradiated field: Case report and review of the literature. Radiat. Oncol..

[15] Bonomo P (2017). Incidence of skin toxicity in squamous cell carcinoma of the head and neck treated with radiotherapy and cetuximab: A systematic review. Crit. Rev. Oncol. Hematol..

[16] Song, P.N., *et al*. Modulation of the tumor microenvironment with trastuzumab enables radiosensitization in HER2+ breast cancer. *Cancers (Basel)*. **14**(4) (2022). 10.3390/cancers14041015 PMC8869800 35205763

[17] Hormuth, D.A., *et al*. Towards an image-informed mathematical model of in vivo response to fractionated radiation therapy. *Cancers (Basel)*. **13**(8) (2021). 10.3390/cancers13081765 PMC8067722 33917080

[18] Virostko J (2021). Quantitative multiparametric MRI predicts response to neoadjuvant therapy in the community setting. Breast Cancer Res..

[19] Sorace AG (2017). Quantitative [(18)F]FMISO PET imaging shows reduction of hypoxia following trastuzumab in a murine model of HER2+ breast cancer. Mol. Imaging Biol..

[20] Reeves, K.M., *et al*. 18F-FMISO PET imaging identifies hypoxia and immunosuppressive tumor microenvironments and guides targeted Evofosfamide therapy in tumors refractory to PD-1 and CTLA-4 inhibition. *Clin. Cancer Res*. (2021). 10.1158/1078-0432.CCR-21-2394 PMC8776604 34615724

[21] Lu, Y., *et al*. [(89)Zr]-Pertuzumab PET imaging reveals paclitaxel treatment efficacy is positively correlated with HER2 expression in human breast cancer xenograft mouse models. *Molecules*. **26**(6) (2021). 10.3390/molecules26061568 PMC8001650 33809310

[22] Jarrett AM (2019). Mathematical modelling of trastuzumab-induced immune response in an in vivo murine model of HER2+ breast cancer. Math. Med. Biol..

[23] Syed, A.K., *et al*. Multiparametric analysis of longitudinal quantitative MRI data to identify distinct tumor habitats in preclinical models of breast cancer. *Cancers (Basel)*. **12**(6) (2020). 10.3390/cancers12061682 PMC7352623 32599906

[24] Syed AK (2019). Characterizing trastuzumab-induced alterations in intratumoral heterogeneity with quantitative imaging and immunohistochemistry in HER2+ breast cancer. Neoplasia.

[25] Barnes, S.L., *et al*. DCE- and DW-MRI as early imaging biomarkers of treatment response in a preclinical model of triple negative breast cancer. *NMR Biomed*. **30**(11) (2017). 10.1002/nbm.3799 28915312

[26] Sorace AG (2016). Trastuzumab improves tumor perfusion and vascular delivery of cytotoxic therapy in a murine model of HER2+ breast cancer: Preliminary results. Breast Cancer Res. Treat..

[27] Song PN (2020). CD4 T-cell immune stimulation of HER2+ breast cancer cells alters response to trastuzumab in vitro. Cancer Cell Int..

[28] Demidenko E, Miller TW (2019). Statistical determination of synergy based on Bliss definition of drugs independence. PLoS One.

[29] Ebert K (2020). Determining the effects of trastuzumab, cetuximab and afatinib by phosphoprotein, gene expression and phenotypic analysis in gastric cancer cell lines. BMC Cancer.

[30] Assenat E (2015). Dual targeting of HER1/EGFR and HER2 with cetuximab and trastuzumab in patients with metastatic pancreatic cancer after gemcitabine failure: Results of the "THERAPY"phase 1–2 trial. Oncotarget.

[31] Lindberg JM (2014). Co-treatment with panitumumab and trastuzumab augments response to the MEK inhibitor trametinib in a patient-derived xenograft model of pancreatic cancer. Neoplasia.

[32] Larbouret C (2010). Combined cetuximab and trastuzumab are superior to gemcitabine in the treatment of human pancreatic carcinoma xenografts. Ann. Oncol..

[33] Zheng L (2014). Combining trastuzumab and cetuximab combats trastuzumab-resistant gastric cancer by effective inhibition of EGFR/ErbB2 heterodimerization and signaling. Cancer Immunol. Immunother..

[34] Erjala K (2006). Signaling via ErbB2 and ErbB3 associates with resistance and epidermal growth factor receptor (EGFR) amplification with sensitivity to EGFR inhibitor gefitinib in head and neck squamous cell carcinoma cells. Clin. Cancer Res..

[35] Wheeler DL (2008). Mechanisms of acquired resistance to cetuximab: Role of HER (ErbB) family members. Oncogene.

[36] Huang F (2019). Simultaneous inhibition of EGFR and HER2 via afatinib augments the radiosensitivity of nasopharyngeal carcinoma cells. J. Cancer.

[37] Kazerouni, A.S., *et al*. Quantifying tumor heterogeneity via MRI habitats to characterize microenvironmental alterations in HER2+ breast cancer. *Cancers (Basel)*. **14**(7) (2022). 10.3390/cancers14071837 PMC8997932 35406609

[38] Bloom MJ (2020). Anti-HER2 induced myeloid cell alterations correspond with increasing vascular maturation in a murine model of HER2+ breast cancer. BMC Cancer.

[39] Ritter CA (2007). Human breast cancer cells selected for resistance to trastuzumab in vivo overexpress epidermal growth factor receptor and ErbB ligands and remain dependent on the ErbB receptor network. Clin. Cancer Res..

[40] Gutsch, D., *et al*. Inhibition of HER receptors reveals distinct mechanisms of compensatory upregulation of other HER family members: Basis for acquired resistance and for combination therapy. *Cells*. **10**(2) (2021). 10.3390/cells10020272 PMC7911202 33572976

[41] Zhang N (2016). HER3/ErbB3, an emerging cancer therapeutic target. Acta Biochim. Biophys. Sin. (Shanghai).

[42] Yonesaka K (2019). Aberrant HER3 ligand heregulin-expressing head and neck squamous cell carcinoma is resistant to anti-EGFR antibody cetuximab, but not second-generation EGFR-TKI. Oncogenesis.

[43] Brand TM (2013). Mapping C-terminal transactivation domains of the nuclear HER family receptor tyrosine kinase HER3. PLoS One.

[44] Zeng L (2020). CHK1/2 inhibitor prexasertib suppresses NOTCH signaling and enhances cytotoxicity of cisplatin and radiation in head and neck squamous cell carcinoma. Mol. Cancer Ther..

[45] Hatakeyama H (2010). Regulation of heparin-binding EGF-like growth factor by miR-212 and acquired cetuximab-resistance in head and neck squamous cell carcinoma. PLoS One.

