# Supplementary material for: Dual anti-HER2/EGFR inhibition synergistically increases therapeutic effects and alters tumor oxygenation in HNSCC
Source: Sci Rep. 2024 Feb 14;14:3771. doi: 10.1038/s41598-024-52897-5 (PMC10866896; doi:10.1038/s41598-024-52897-5)
Supplement: Supplementary file 1 — Supplementary Legends. [file 41598_2024_52897_MOESM1_ESM.docx]

**Supplementary Table 1: Treatment dosing strategy for *in vitro* experiments**
