# Supplementary material for: Dual anti-HER2/EGFR inhibition synergistically increases therapeutic effects and alters tumor oxygenation in HNSCC
Source: Sci Rep. 2024 Feb 14;14:3771. doi: 10.1038/s41598-024-52897-5 (PMC10866896; doi:10.1038/s41598-024-52897-5)
Supplement: Supplementary file 2 — Supplementary Table 1. [file 41598_2024_52897_MOESM2_ESM.pdf]

In vitro **experiments**	**Trastuzumab**		**Cetuximab**		**Radiation**
Dose	50 µg/mL	100 µg/mL	250 µg/mL	500 µg/mL	2 Gy
Experiment 2.3.1		X	X		
Experiment 2.3.2	X	X	X	X	
Experiment 2.3.3		X	X		X
Experiment 2.3.4		X	X		X
Experiment 2.3.5		X	X		X
Experiment 2.3.6		X	X		X
